# Sclerosing Lymphocytic Lobulitis Mimicking a Tumor Relapse in a Young Woman with a History of Breast Cancer

**DOI:** 10.5334/jbr-btr.835

**Published:** 2015-09-15

**Authors:** J. Decraene, C. Van Ongeval, G. Clinckemaillie, H. Wildiers

**Affiliations:** 1 Department of Radiology, Universitaire Ziekenhuizen Leuven, Leuven, Belgium; 2 Department of Oncology, Universitaire Ziekenhuizen Leuven, Leuven, Belgium

**Keywords:** Breast diseases

## Abstract

Sclerosing lymphocytic lobulitis or diabetic mastopathy is a benign entity with non-specific imaging features which can mimic breast carcinoma. It is a condition commonly associated with long standing diabetes and has also been linked with various auto-immune diseases. We present the case of a 27-year-old woman with a history of carcinoma of the left breast and otherwise unremarkable medical history, who developed sclerosing lymphocytic lobulitis in the right breast during follow-up.

## Case report

We present the case of a 27-year-old woman who was referred to our hospital for further investigation, after worrying findings during a routine check-up performed in another hospital. Five years prior to this check-up, the patient was diagnosed with cancer of the left breast at the very young age of 22. The tumor was staged as pT2N0M0, with the histologic examination showing a poorly differentiated invasive ductal adenocarcinoma with strong estrogen and progesterone receptor expression and negative herceptin status. The treatment consisted of a wide excision and sentinel node procedure, followed by adjuvant chemotherapy, radiotherapy and hormonal therapy. Because of her young age, 3 cycles of cyclophosphamide-epirubicin-fluorouracil (FEC) and 3 cycles of docetaxel were given. Chemotherapy was followed by radiotherapy of the left breast up to a dose of 50 Gy, with a boost of 16 Gy on the tumor bed. Hormonal therapy consisted of a combination of tamoxifen and triptorelin. There was no relevant personal medical history, nor family history of breast cancer. Genetic analysis failed to show any BRCA1 or BRCA2 mutations.

**Figure 1:**
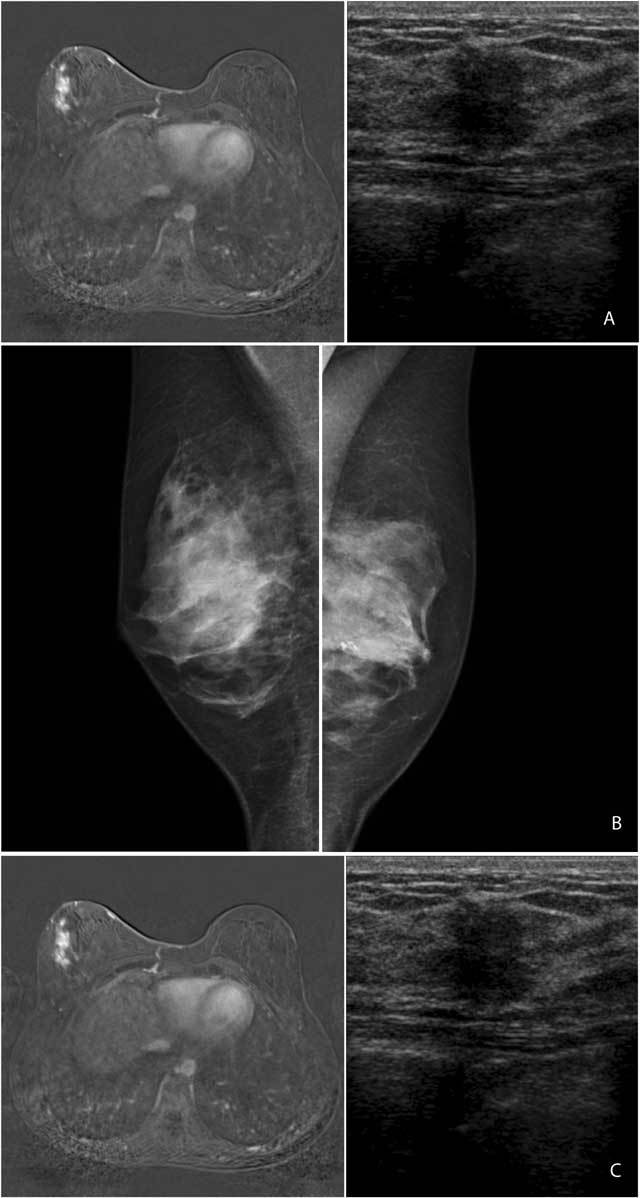
2012: Mammogram of the right breast shows no peculiarities; in the left breast there is a macrocalcification due to liponecrosis due to surgery (A). MRI of the breasts, T1 substraction image after contrast administration shows ill-defined multicentric nodular contrast enhancement in the left breast (B). Ultrasound of this region shows an ill-defined hypoechoic mass with mild posterior acoustic shadowing (C).

The patient recovered well and follow-up examinations were normal. At the time of the check-up, five years after surgery, coinciding with the conclusion of the hormonal therapy, the follow-up mammogram (Fig. 1A) and first ultrasound showed the surgery related changes. (Fig. 1A) Because of her young age, magnetic resonance imaging (MRI) of the breasts was also performed (examination performed on Siemens Magnetom Symphony 1.5T), showing a multifocal nodular contrast enhancement in the retro-areolar region and the lower-outer quadrant of the right breast (Fig. 1B). These findings were not present on the previous MRI, performed two years after surgery. There were no clinical abnormalities in the region of this contrast enhancement. Because of the unclear etiology of these findings, the woman was referred to our department for further investigation. On ultrasonography, we were able to visualize some parenchymal distortion and an ill-defined hypoechoic lesion with a diameter around 1 cm and mild posterior acoustic shadowing in the region of the contrast enhancement on MRI (Fig. 1C). Because of the ultrasound and MRI findings and the patient’s history of breast cancer, the examinations were categorized as BIRADS 4. An ultrasound-assisted core biopsy (4 × 14 Gauge) was performed. Histologic examination showed a dense lymphocytic infiltrate of predominantly B-lymphocytes surrounding the ductulolobular unit and the vessels, in combination with a fibrous stroma of low cellularity and an increase in fibroblasts. These findings led to a diagnosis of sclerosing lymphocytic lobulitis. No signs of malignancy were detected.

**Figure 2:**
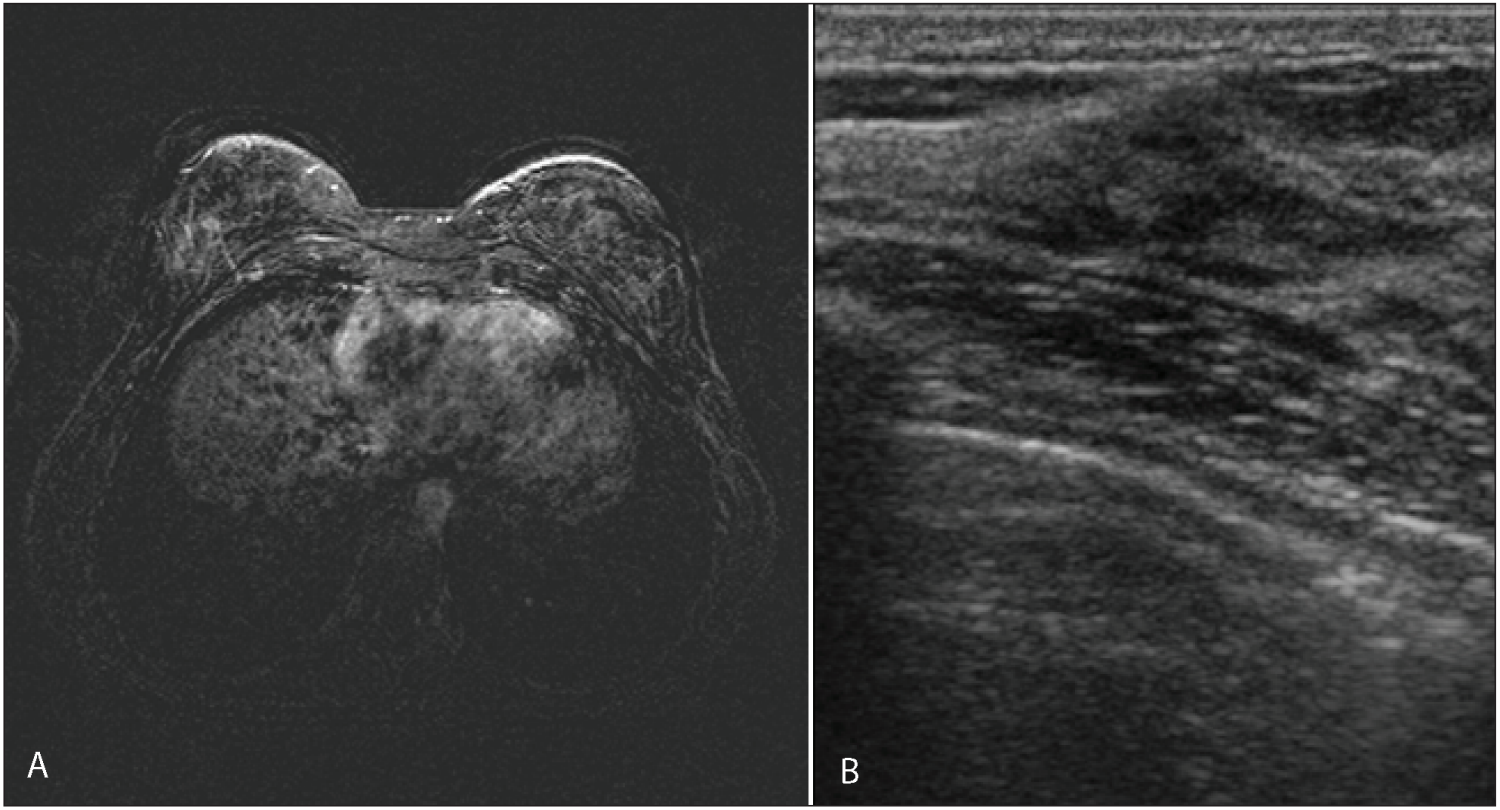
2014: MRI of the breasts, T1 substraction image after contrast administration shows a marked decrease in contrast enhancement (A). On ultrasound the volume and acoustic shadowing of the lesion has also decreased (B).

After diagnosis of sclerosing lymphocytic lobulitis, follow-up consisted of a breast ultrasound every six months and a yearly mammogram and MRI of the breasts. The volume of the hypoechoic lesion on ultrasonography and the intensity of the gradual multifocal contrast enhancement on MRI have both progressively diminished on consecutive examinations, to a level where it is barely perceptible (Fig. 2A and B).

## Discussion

Sclerosing lymphocytic lobulitis (SLL) has been reported in the literature using various different designations, of which ‘diabetic mastopathy’ is another commonly used term. The entity has long been regarded as a complication of long-standing diabetes, often in cases where the diabetes is poorly regulated and in the presence of other diabetic complications. The association between diabetes and breast disease was first described by Soler and Khardori in 1984, and they were the first to suggest a role of autoimmunity in the pathogenesis. SLL has indeed also been reported in combination with auto-immune diseases such as Hashimoto thyroiditis and Sjogren’s syndrome. In some cases the auto-immune disease only developed after the diagnosis of SLL.

The pathogenesis is unclear and is thought to be related to a deposition of non-enzymatically glycosylated proteins within the extracellular matrix, inducing a local immunogenic response. In turn, this would lead to a perivascular proliferation of B-lymphocytes and inflammation of the lobular epithelium, as well as matrix expansion and proliferation of fibroblasts.

Typically, the patient presents with a firm to hard, painless and freely movable breast lump. The lesions can be multicentric or bilateral, both synchronous or metachronous.

Mammograms in patients with SLL show often but not always a regional asymmetric increased opacity with ill-defined margins, without microcalcifications or overlying skin changes. The lesions are often occult in dense breasts. Typical ultrasonography findings include a hypoechoic mass with indistinct margins and posterior acoustic shadowing. Data on the magnetic resonance imaging findings in confirmed cases of SLL are rather scarce, although most authors describe a regional, gradually increasing stromal contrast enhancement in absence of a focal enhancing mass, with a time-intensity curve more suited to a benign lesion, which might help differentiating SLL from malignancy.

It is widely reported that SLL is neither premalignant nor malignant and the entity should therefore be managed as such. However, radiologists must be aware that the imaging findings of SLL are nonspecific and that core biopsy is needed to exclude malignancy. Fine needle aspiration cytology is not recommended because of the low cellularity of the condition.

As for therapy, SLL in itself requires no treatment, but of course any underlying diabetes or auto-immune disease should be managed accordingly. Surgery has been performed in some cases, but should be restricted to symptomatic patients, as lesions tend to recur after resection. Once malignancy has been excluded, no specific follow-up is needed, and the routine breast cancer screening schedule can be resumed.

This patient did not have diabetes or any known auto-immune diseases and repeated glycemia and HbA1c checks were normal. We were unable to find another case report of a non-diabetic patient with breast carcinoma in one breast and sclerosing lymphocytic lobulitis in the other breast. A consideration for the possible etiology of the SLL in our case could be that the local tissue destruction caused by surgery and chemotherapy triggered an auto-immune response against the patient’s own breast tissue, although she was only diagnosed with SLL after conclusion of the hormonal therapy.

## Competing Interests

The authors declare that they have no competing interests.
